# Urinary metabolic profile and stone composition in kidney stone formers with and without heart disease

**DOI:** 10.1007/s40620-021-01096-w

**Published:** 2021-06-21

**Authors:** Matteo Bargagli, Shabbir Moochhala, William G. Robertson, Giovanni Gambaro, Gianmarco Lombardi, Robert J. Unwin, Pietro Manuel Ferraro

**Affiliations:** 1 U.O.S. Terapia Conservativa della Malattia Renale Cronica, U.O.C. Nefrologia, Fondazione Policlinico Universitario A. Gemelli IRCCS, Università Cattolica del Sacro Cuore, Largo Agostino Gemelli 8, 00168 Rome, Italy; 2 Department of Renal Medicine, Royal Free Campus Medical School, University College Hospital, London, UK; 3 Nuffield Department of Surgical Sciences, University of Oxford, Oxford, UK; 4 Renal Unit, Division of Nephrology and Dialysis, Department of Medicine, University of Verona, Verona, Italy; 5 Dipartimento Universitario di Medicina e Chirurgia Traslazionale, Università Cattolica del Sacro Cuore, Rome, Italy

**Keywords:** Kidney stones, Cardiovascular risk, Magnesium, Citrate

## Abstract

**Objective:**

Kidney stone disease seems to be associated with an increased risk of incident cardiovascular outcomes; the aim of this study is to identify differences in 24-h urine excretory profiles and stone composition among stone formers with and without cardiovascular disease (CVD).

**Methods:**

Data from patients attending the Department of Renal Medicine’s metabolic stone clinic from 1995 to 2012 were reviewed. The sample was divided according to the presence or absence of CVD (myocardial infarction, angina, coronary revascularization, or surgery for calcified heart valves). Univariable and multivariable regression models, adjusted for age, sex, BMI, hypertension, diabetes, eGFR, plasma bicarbonate and potential renal acid load of foods were used to investigate differences across groups.

**Results:**

1826 patients had available data for 24-h urine analysis. Among these, 108 (5.9%) had a history of CVD. Those with CVD were older, have higher prevalence of hypertension and diabetes and lower eGFR. Univariable analysis showed that patients with CVD had significantly lower 24-h urinary excretions for citrate (2.4 vs 2.6 mmol/24 h, p = 0.04), magnesium (3.9 vs 4.2 mmol/24 h, p = 0.03) and urinary pH (6.1 vs 6.2, p = 0.02). After adjustment for confounders, differences in urinary citrate and magnesium excretions remained significant. No differences in the probability of stone formation or stone compositions were found.

**Conclusions:**

Stone formers with CVD have lower renal alkali excretion, possibly suggesting higher acid retention in stone formers with cardiovascular comorbidities. Randomized clinical trials including medications and a controlled diet design are needed to confirm the results presented here.

**Graphic abstract:**

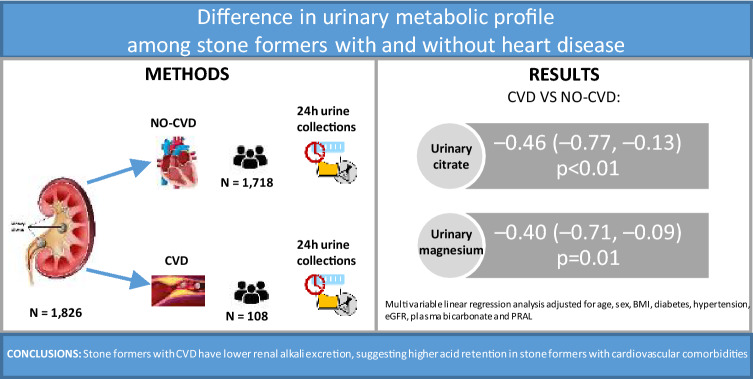

## Introduction

Nephrolithiasis is a medical condition with a high prevalence in the general population in Europe and the United States [1, 2]. Kidney stone disease is a significant clinical and financial burden with an annual expenditure of up to $10 billion in the United States alone [3]. The last decades have been characterized by an increase in the incidence of nephrolithiasis associated with a progressive reduction in the male to female ratio, especially in the United States [4]. This may be explained by changes in lifestyle and nutritional habits, leading to more obesity in women, which is a known risk factor for kidney stones. In addition to obesity [5], nephrolithiasis is associated with other comorbidities such as arterial hypertension [6], diabetes mellitus [7] and metabolic syndrome [8]. Nephrolithiasis has also been linked to an increased likelihood of developing chronic kidney disease (CKD) and of cardiovascular events [9], suggesting that kidney stone disease is a systemic disorder, but the reasons for these associations is still unknown.

Recent studies have revealed that kidney stone patients have an increased incidence of specific cardiovascular anomalies such as an augmented pulse-wave velocity, arterial stiffness [10] and vascular calcification [11] when compared with the general population. However, data supporting the link between kidney stones disease and cardiovascular outcomes are from epidemiological studies, and it could be that certain 24-h urine abnormalities are associated with a greater risk of cardiovascular disease (CVD).

Higher urinary citrate excretion prevents lithogenesis, inhibiting the aggregation of calcium-oxalate crystals and neutralizing uric acid supersaturation. Hypocitraturia is a common finding in kidney stone-formers [12] and is also associated with a higher prevalence of abdominal aortic calcification (AAC) [13].

Another mineral involved in kidney stone formation is magnesium. This ion reduces both crystallization and growth of calcium-oxalate stones, so that lower urinary excretion and dietary deficiency are considered risk factors for nephrolithiasis [14]. Furthermore, lower serum magnesium levels have been associated with all-cause mortality, cardiovascular mortality and vascular calcifications in CKD and hemodialysis populations [15, 16]. Based on this evidence, a recent randomized trial demonstrated the effectiveness of magnesium-oxide supplementation in slowing progression of coronary artery calcification [17].

In light of these observations, the aim of the present study was to investigate differences in 24-h urine excretory profiles and stone composition in stone-formers with and without CVD.

## Methods

### Study population and data collection

We performed a retrospective observational analysis of data from patients attending the UCL Department of Renal Medicine’s metabolic stone clinic from 1995 to 2012. Details of the cohort have been reported elsewhere [12]. We included all adult (≥ 18 years old) patients with at least one metabolic assessment of nephrolithiasis (24-h urine analysis). For each patient, demographic, and clinical information (sex, age, body mass index (BMI), comorbidities) were recorded. Cardiovascular disease was defined as the presence of one or more of the following self-reported conditions: myocardial infarction, angina, coronary revascularization, or surgery for calcified heart valves. All included patients performed a fasting blood sample for serum sodium, calcium, magnesium, phosphate, creatinine, plasma bicarbonate, and a 24-h urine collection for measurement of urine volume, urine pH and 24-h urinary excretion of the following solutes: creatinine, calcium, oxalate, urate, citrate, magnesium, sodium and potassium. Patients also underwent a food frequency questionnaire for dietary determination of daily fluids, sodium, potassium, calcium, magnesium, phosphate, animal protein and fiber intakes. Nutrients consumption from food frequency questionnaires were estimated through a validated software based on McCance and Widdowson’s Food Composition Tables [18].

Glomerular filtration rate was estimated (eGFR) using the Chronic Kidney Disease Epidemiology Collaboration equation [19].

Potential renal acid load of foods was calculated as follow: (0.49 * protein intake) + (0.037 × phosphate intake) – (0.021 × potassium intake) – (0.026 × magnesium intake) – (0.013 × calcium intake)[20].

Hypercalciuria was defined as urine calcium excretion > 6.2 mmol/24-h for women and 7.5 mmol/24-h for men. Hyperuricosuria was considered as urinary uric acid excretion > 4.5 mmol/24-h for females and > 4.8 mmol/24-h for males. Hyperoxaluria was defined as urinary oxalate excretion > 0.5 mmol/24-h for both males and females. Hypocitraturia was considered as urinary citrate excretion < 1.5 mmol/24-h. Low urine volume was considered as urine volume < 1 L/24-h.

Stone compositions were considered to have a single constituent if a proportion greater than 95% of the stone weight was composed of a single component.

A validated calculation for the risk of stone formation (computation of the probability of stone formation or PSF) was obtained for calcium oxalate, calcium phosphate and uric acid in all patients [21].

### Statistical analysis

Continuous variables were reported as means and standard deviations, categorical variables as frequencies and percentages. Differences in 24-h urine composition between stone formers with and without CVD were investigated using Mann-Whitney test, Fisher exact test, univariable and multivariable regression models, adjusted for age, sex, BMI, diabetes, hypertension, eGFR, plasma bicarbonate and PRAL. A p-value ≤ 0.05 was considered as statistically significant. All analyses were performed with Stata version 16 (StataCorp, Texas, US).

## Results

A total of 1826 patients had available serum parameters and 24-h urine collections and were included in the analysis. Among these, 108 (5.9 %) subjects had history of CVD. Those with CVD were older (59 vs. 46 years, p < 0.01), whereas BMI (27.4 vs. 26.7, p = 0.13) and sex was similar (males, 73 vs. 70 %, p = 0.56) between groups. Stone formers affected by CVD have higher prevalence of hypertension and diabetes and lower eGFR (Table 1). Overall, hypercalciuria was the most common urinary abnormalities (n = 658, 36 %), followed by hypocitraturia (n = 409, 22 %), hyperuricosuria (n = 310, 17 %), hyperoxaluria (n = 151, 8.3 %) and low urine volume (n = 103, 5.6 %). Univariable analyses showed that patients with CVD had significantly lower urinary excretions for citrate (2.4 vs. 2.6 mmol/24-h, p = 0.04) and magnesium (3.9 vs. 4.2 mmol/24-h, p = 0.03) associated to lower 24-h urine pH (6.1 vs. 6.2, p = 0.02) and increased prevalence for hypocitraturia (32.4 % vs. 21.8 %, p = 0.01) (Table 1). Dietary sodium intake was significantly lower in CVD stone formers (170 vs. 176 mmol/day, p = 0.04) (Table 2). No differences in the remaining dietary component, serum parameters or PSF for calcium oxalate, brushite or uric acid were found between study groups.

**Table 1:** Baseline characteristics by CVD status

Variables	All patients (N = 1826)	No CVD stone formers (N = 1718)	CVD stone formers (N = 108)	p-value
Age, years	47.2 (13.7)	46.5 (13.4)	58.8 (12.9)	< 0.001
Females	544 (29.8)	515 (30.0)	29 (26.9)	0.562
Hypertension	325 (17.8)	294 (17.1)	31 (28.7)	0.012
Diabetes	118 (6.5)	103 (6.0)	15 (14.9)	0.009
BMI	26.7 (4.9)	26.7 (4.9)	27.4 (4.3)	0.133
eGFR, mL/min/1.73m^2^, CKD-EPI	84.1 (21.5)	85.1 (21.2)	69.7 (20.7)	< 0.001
Serum sodium, mmol/L	141.5 (2.2)	141.5 (2.2)	141.4 (2.5)	0.604
Serum potassium, mmol/L	4.30 (0.41)	4.31 (0.40)	4.27 (0.46)	0.383
Plasma bicarbonate, mmol/L	27.6 (3.0)	27.6 (3.0)	27.6 (3.0)	0.902
Serum calcium, mmol/L	2.42 (0.13)	2.42 (0.12)	2.43 (0.14)	0.391
Serum phosphate, mmol/L	1.07 (0.19)	1.07 (0.19)	1.04 (0.18)	0.092
Serum magnesium, mmol/L	0.83 (0.07)	0.83 (0.07)	0.83 (0.08)	0.186
Urine citrate, mmol/day	2.63 (1.38)	2.64 (1.38)	2.36 (1.48)	0.042
Urine potassium, mmol/day	70.8 (22.0)	70.8 (22.1)	71.2 (21.3)	0.839
Urine magnesium, mmol/day	4.21 (1.34)	4.22 (1.34)	3.93 (1.31)	0.026
Urine oxalate, mmol/day	0.35 (0.29, 0.41)	0.35 (0.29, 0.41)	0.35 (0.30, 0.41)	0.638
Urine calcium, mmol/day	6.39 (3.05)	6.42 (3.02)	5.84 (3.47)	0.053
Urine uric acid, mmol/day	3.63 (1.18)	3.64 (1.18)	3.49 (1.13)	0.191
Urine creatinine, mmol/day	13.3 (3.5)	13.3 (3.5)	12.9 (3.1)	0.178
Urine sodium, mmol/day	164 (60)	164 (61)	154 (52)	0.078
Urine pH	6.20 (0.58)	6.20 (0.58)	6.07 (0.52)	0.020
Urine volume, L/day	1.93 (1.45, 2.54)	1.93 (1.45, 2.55)	1.99 (1.45, 2.51)	0.918
Low urine volume	103 (5.6)	96 (5.6)	7 (6.5)	0.861
Hypocitraturia	409 (22.4)	374 (21.8)	35 (32.4)	0.014
Hypercalciuria	658 (36.0)	626 (36.4)	32 (29.6)	0.185
Hyperuricosuria	310 (17.0)	295 (17.2)	15 (13.9)	0.454
Hyperoxaluria	151 (8.3)	145 (8.4)	6 (5.6)	0.381
PSF CaOx	0.43 (0.33)	0.43 (0.34)	0.41 (0.33)	0.643
PSF CaPi	0.54 (0.30)	0.54 (0.29)	0.49 (0.33)	0.111
PSF UA	0.04 (0.17)	0.04 (0.17)	0.05 (0.18)	0.670

Categorical variables are reported as frequencies (%). Continuous variables are reported as means (SD) or medians (25th, 75th percentile)

*CVD* cardiovascular disease, *eGFR* estimated glomerular filtration rate, *PSF* probability of stone formation

**Table 2:** Dietary intake by CVD status

Variables	All patients (N = 1561)	No CVD stone formers (N = 1455)	CVD stone formers (N = 106)	p-value
Fluid intake, L/day	2.66 (2.18, 3.27)	2.67 (2.18, 3.28)	2.53 (2.07, 3.17)	0.105
Sodium intake, mmol/day	176.0 (146.0, 217.0)	176.0 (146.0, 218.0)	170.0 (132.0, 204.0)	0.043
Potassium intake, mmol/day	83.2 (20.6)	83.3 (20.7)	82.8 (19.4)	0.822
Calcium intake, mmol/day	22.1 (18.4, 26.9)	22.2 (18.4, 26.9)	20.8 (17.2, 27.3)	0.139
Magnesium intake, mmol/day	15.5 (4.0)	15.5 (4.0)	14.9 (4.2)	0.176
Phosphate intake, mmol/day	45.3 (38.6, 52.2)	45.4 (38.6, 52.3)	43.5 (37.9, 51.8)	0.365
Oxalate intake, mmol/day	2.09 (1.64, 2.65)	2.09 (1.64, 2.65)	2.08 (1.53, 2.76)	0.462
Animal protein intake, g/day	55.9 (18.9)	56.0 (19.0)	54.9 (17.0)	0.575
Fiber intake, g/day	21.0 (17.0, 25.4)	20.9 (16.9, 25.4)	21.0 (17.1, 25.7)	0.839
PRAL, mEq/day	– 10.3 (16.5)	–10.3 (16.6)	– 10.5 (16.5)	0.925

Continuous variables are reported as means (SD) or medians (25th, 75th percentile)

*CVD* cardiovascular disease, *PRAL* potential renal acid load

A subgroup of 677 patients had available data for stone composition analysis: the proportion of calcium oxalate (57 vs. 53 %, p = 0.54), calcium phosphate (19 vs. 28 %, p = 0.09), and uric acid (16 vs. 12 %, p = 0.35) in analyzed stones was similar for those with and without CVD (Table 3).

**Table 3:** Stone composition by CVD status

Variables	All patients (N = 677)	No CVD stone formers (N = 631)	CVD stone formers (N = 46)	p-value
CaOx stones	0.53 (0.40)	0.53 (0.40)	0.57 (0.41)	0.543
CaPi stones	0.27 (0.32)	0.28 (0.33)	0.19 (0.27)	0.089
UA stones	0.12 (0.31)	0.12 (0.31)	0.16 (0.36)	0.345

Continuous variables are reported as means (SD)

*CaOx* calcium oxalate, *CaPi* calcium phosphate, *UA* uric acid, *CVD* cardiovascular disease

After adjustment for age, sex, BMI, diabetes, hypertension, eGFR, plasma bicarbonate and PRAL, urinary uric acid, citrate and magnesium excretions were significantly lower in CVD stone formers, compared with those without heart disease (β: − 0.25, p = 0.05; β: − 0.46, p < 0.01; β: − 0.40, p = 0.01, respectively) (Table 4). The results were unchanged after removing 177 participants with urine creatinine below or above the sex-specific 5th and 95th percentile.

**Table 4:** Multivariable regression models for CVD status

	N of observations	β coefficient	95 % confidence interval	p-value
Serum sodium, mmol/L	1201	0.05	– 0.48, 0.59	0.845
Serum potassium, mmol/L	1197	– 0.01	– 0.11, 0.08	0.784
Serum calcium, mmol/L	1201	– 0.01	– 0.05, 0.02	0.400
Serum phosphate, mmol/L	1197	– 0.02	– 0.07, 0.02	0.346
Serum magnesium, mmol/L	1196	– 0.001	– 0.02, 0.02	0.894
Urine citrate, mmol/day	1201	– 0.45	– 0.77, − 0.13	0.006
Urine potassium, mmol/day	1201	– 2.79	– 7.68, 2.10	0.264
Urine magnesium, mmol/day	1201	– 0.40	– 0.71, − 0.09	0.012
Urine oxalate, mmol/day^a^	1201	– 0.05	– 0.11, 0.02	0.184
Urine calcium, mmol/day	1201	– 0.54	– 1.26, 0.18	0.140
Urine uric acid, mmol/day	1201	– 0.25	– 0.50, − 0.003	0.047
Urine sodium, mmol/day	1201	– 10.37	– 24.10, 3.36	0.139
Urine pH	1201	0.09	– 0.03, 0.20	0.150
Urine volume, L/day^a^	1201	– 0.03	– 0.13, 0.06	0.504

Multivariable linear regression analysis between CVD status and serum and urinary parameters, adjusted for age, sex, BMI, diabetes, hypertension, eGFR, plasma bicarbonate and PRAL

*CVD* cardiovascular disease

^a^Natural logarithm transformed

## Discussion

Kidney stone disease is known to be associated with an increased risk of myocardial infarction and cardiovascular events [22]; moreover, both coronary heart disease and vascular calcification are common findings in patients with kidney stones and CKD [11, 23]. Nephrolithiasis itself is associated with atheroma and vascular calcification: Reiner et al. found an already increased prevalence of carotid artery atherosclerosis in a population of young (aged 18–30 years) patients with a history of kidney stone disease [24]. A subsequent meta-analysis of 11 studies confirmed this finding; nephrolithiasis seems to be associated with an increased cardiovascular risk, especially coronary heart disease and stroke [25].

However, these associations appear to be valid for only some kidney stone phenotypes: we found previously a direct association between calcium phosphate content of stones and the presence of AAC (Odds Ratio (OR) 1.25, 95 % CI 1.00, 1.56, p = 0.045) [26]. Despite this, the pathophysiological mechanisms underlying the epidemiological association between cardiovascular disease and kidney stone risk are still unresolved.

In the present study, 5.9 % of patients were affected by cardiovascular disease. In the univariable analysis, urine pH, urinary citrate and magnesium excretions were significantly lower in CVD stone formers, when compared with patients without cardiovascular risk factors. CVD stone formers appear to be older, have a lower eGFR and a higher prevalence of diabetes and hypertension. However, after adjusting for potential confounders, including age and sex, BMI, diabetes, hypertension, eGFR, plasma bicarbonate and PRAL, differences in urinary excretions of citrate and magnesium remained statistically significant between the study groups.

A common pathway linking CVD and kidney stones is hypocitraturia. A recent retrospective analysis of 97 stone formers showed the association between AAC and lower urinary citrate excretion compared with controls (399 vs. 593 mg/24-h, p < 0.001). Furthermore, after adjusting for confounders, AAC was found to be associated with hypocitraturia (< 320 mg/24-h, OR 4.37, p = 0.005) [13]. Low urinary rcitrate excretion is one of the most common 24-h urine abnormalities found in calcium phosphate stone formers [26].

Metabolic syndrome, type 2 diabetes and underlying insulin resistance are well known risk factors for both cardiovascular events and uric acid nephrolithiasis, a kidney stone phenotype characterized by a more acidic urine pH and hypocitraturia [8, 27, 28]. However, in the present study, after adjusting for diabetes and BMI, urinary citrate excretion remained significantly different across groups, possibly indicating that a different mechanism, other than pH, is involved. In addition, lower urinary citrate excretion was accompanied by similar plasma bicarbonate concentrations, dietary alkali intake and PRAL of foods, between stone formers with and without cardiovascular disease. Since urinary citrate excretion has been found recently to be superior to plasma bicarbonate concentration in detecting latent metabolic acidosis in CKD patients [29, 30], our results may s indicate higher acid retention in stone-formers with cardiovascular comorbidities.

To try to explain our results, we reviewed the role of magnesium in prevention of CVD. Magnesium is critical in both cardiac remodeling, myocardial contraction and relaxation [15], and in the inhibition of calcium-oxalate crystallization [14]. Furthermore, the main regulator of magnesium balance is not bone turnover or gastrointestinal uptake, but its renal handling [31]. However, the effect of reduced urinary magnesium excretion on clinical outcomes seems to be controversial: a higher risk for CVD was observed in those with reduced urinary magnesium excretion in a general population, although plasma magnesium concentrations or dietary intake were not provided[32]. More recently, after adjusting for several potential confounders, a positive association between 24-h urinary magnesium and cardiovascular events was found in patients with CKD and normal serum magnesium levels [33]. This may be related to an impaired ability of the kidney to completely reabsorb the filtered load of magnesium in CKD.

As already described, lower serum magnesium levels are associated with a higher risk for cardiovascular events and magnesium-containing supplements can slow progression of coronary artery calcification in CKD patients [17]. However, in our investigation, lower urinary magnesium excretion was observed despite similar dietary magnesium intake and serum magnesium levels between study groups. Of note, a slightly lower urinary uric acid excretion was noted in CVD stone-formers. Diuretics are known to alter the renal excretion of citrate, magnesium and uric acid[34–36] and our findings may, at least in part, be influenced by differences in medical treatment across the groups.

This study has several limitations, including incomplete data on medications, the cross-sectional design, lack of longitudinal information and follow-up, and of exposure validation.

In conclusion, our research is the first to demonstrate that stone-formers affected by heart disease have a multifactorial 24-h urine pattern characterized by lower urinary excretions of both citrate and magnesium. Since citrate and magnesium have been implicated in the pathogenesis of arterial plaque formation, as well as being protective factors in nephrolithiasis, this might indicate a shared underlying pathogenesis. Furthermore, the reduction in renal alkali excretion observed suggests higher acid retention in stone-formers with cardiovascular comorbidities. Since there is a lack of prospective studies, randomized clinical trials that include medications and a controlled diet design are needed to confirm these observations.
